# Silver Staining of Alzheimer's Disease

**DOI:** 10.4172/2329-6895.1000i103

**Published:** 2014-10-28

**Authors:** Orwa Aboud, Sue T. Griffin W

**Affiliations:** 1 Donald W. Reynolds Department of Geriatrics, University of Arkansas for Medical Sciences, Little Rock, AR, USA; 2 Department of Neurology, University of Arkansas for Medical Sciences, Little Rock, AR, USA; 3 Geriatric Research, Education, Clinical Center, Central Arkansas HealthCare System, Little Rock, AR, USA

## Description

Sliver stain of olfactory bulb tissue section in a 74 year old male with a clinical history of Alzheimer’s, congestive heart failure, and pneumonia. A. A 10X magnification for the area containing the anterior olfactory nucleus (AON) (astercs). B. A 20X of the same area showing neurofibrillary tangles (arrow) and neuropil threads (double arrow).

The autopsy showed definite Alzheimer’s disease (C on CERAD scale). Neuritic plaques identified in inferior parietal and parahippocampal areas from this patient. There were moderate tangles in peristriate and frontal cortex, and neuropil threads were prominent in mesial temporal structures and frontal cortex.

Paraffin sections were deparaffinized and hydrated to distilled water, then placed in 20% silver nitrate and kept in 60°C for 15 minutes, then rinsed in distilled water. Then sections were treated with formalin solution containing ammoniacal silver solution for about 15 min, followed by rinsing and placing tissue in sodium thiosulfate solution for 2 minutes and washing in tap water. The section then were dehydrated and mounted with synthetic resin.

## Figures and Tables

**Figure 1A:**
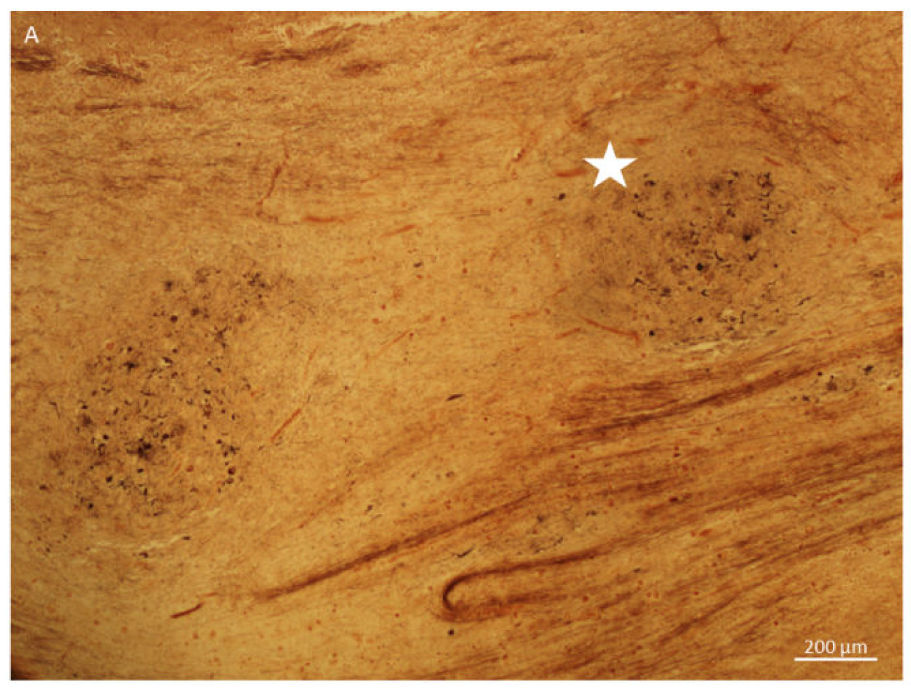
10X magnification for the area containing the anterior olfactory nucleus (AON) (astercs).

**Figure 1B:**
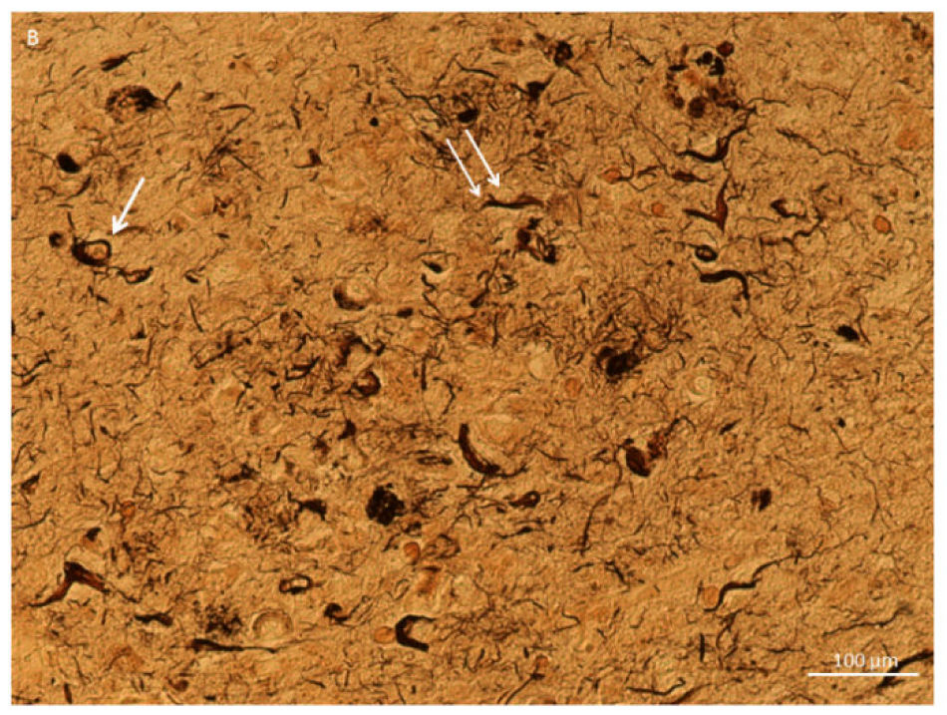
A 20X of the same area showing neurofibrillary tangles (arrow) and neuropil threads (double arrow).

